# The Association Between Adolescent Residential Mobility and Adult Social Anxiety, BDNF and Amygdala-Orbitofrontal Functional Connectivity in Young Adults With Higher Education

**DOI:** 10.3389/fpsyt.2020.561464

**Published:** 2020-12-21

**Authors:** Gregor Hasler, Melanie Haynes, Sabrina Theresia Müller, Ruth Tuura, Christopher Ritter, Andreas Buchmann

**Affiliations:** ^1^Psychiatric University Hospital, University of Bern, Bern, Switzerland; ^2^Unit of Psychiatry Research, University of Fribourg, Fribourg, Switzerland; ^3^Center for MR-Research, University Children's Hospital Zurich, Zurich, Switzerland

**Keywords:** residential mobility, connectivity, fMRI, BDNF, anxiety, stress

## Abstract

**Background:** Large-scale epidemiological studies demonstrate that house moves during adolescence lead to an increase in anxiety and stress-sensitivity that persists into adulthood. As such, it might be expected that moves during adolescence have strong negative and long-lasting effects on the brain. We hypothesized that moves during adolescence impair fear circuit maturation, as measured by the connectivity between amygdala and orbitofrontal cortex, and expression of brain-derived neurotrophic factor (BDNF).

**Methods:** We examined young adults with middle and high economic status recruited from the community using clinical interviews, self-report questionnaires, functional magnetic resonance imaging during an emotional faces task and during a 10 min rest phase, and serum BDNF serum concentration.

**Results:** Out of 234 young adults, 164 did not move between ages 10 and 16 (i.e., moves with change of school), 50 moved once, and 20 moved twice or more than twice. We found relationships between adolescent moving frequency and social avoidance (p_corr_ = 0.012), right amygdala-orbitofrontal cortex connectivity (p_corr_ = 0.016) and low serum BDNF concentrations in young adulthood (p_corr_ = 0.012). Perceived social status of the mother partly mitigated the effects of moving on social avoidance and BDNF in adulthood.

**Conclusions:** This study confirms previous reports on the negative and persistent effects of residential mobility during adolescence on mental health. It suggests that these effects are mediated by impairments in fear circuit maturation. Finally, it encourages research into protecting factors of moving during adolescents such as the perceived social status of the mother.

## Introduction

There is growing evidence that moves, or residential mobility, in childhood and adolescence increase the risk of external behaviors such as impulsive and risky behaviors, and internalizing problems such as stress sensitivity, anxious temperament, and social withdrawal (1).

The impact of residential mobility on child development varies with the age of the child. A Danish national birth cohort study including 1.5 million participants examined moving during each age year between birth and 14 years (2). It demonstrated that the association between moving and health problems increased with age of moving. In addition, it showed that multiple moves during this sensitive time period were associated with worse outcomes than a single move. A follow-up examination of the cohort revealed that mental health problems related to moving in adolescence persisted into adult life.

Adolescence may be a particularly critical period for moving because of the loss of peer networks that are increasingly important in this period. Impairment of peer group socialization is a risk factor for stress-related disorders (3). Moreover, mobile adolescents experience less informal neighborhood control than their stable counterparts (4). As a result, residentially mobile adolescents are more frequently socially isolated, bullied, and affiliated with delinquent peers than residentially stable adolescents, and become less involved in prosocial and achievement-oriented activities (5). Even “upward” moves to wealthier, lower-risk and more prestigious neighborhoods were associated with more frequent school drop-outs (6).

Adolescence is the peak age of many psychiatric problems (7). In this critical phase, there are important changes in brain structure and brain function associated with synaptic reorganization and changes in neuroplasticity (8). Increased plasticity renders the brain highly vulnerable to environmental stress such as moving (9). The connection between the amygdala and the orbitofrontal cortex, a neural substrate of affect regulation, is particularly vulnerable in this period because it undergoes important changes in adolescence (10). The right amygdala-orbitofrontal connectivity is particularly relevant for fear conditioning and anxiety (11, 12).

Brain-derived neurotrophic factor (BDNF) plays a critical role in the development of the prefrontal-amygdala circuit during adolescence (13). Stress-induced reductions in BDNF bioavailability can compromise the integrity of this circuit and increase risk of social deficits. In rodents, chronic social defeat stress led to abnormal expression of BDNF by epigenetic modifications (14). Such modifications may lead to lasting impairments in neuroplasticity, which is widely implicated in psychiatric diseases including anxiety disorders, depression, bipolar disorders, schizophrenia, and addiction (15).

Most previous studies on residential mobility focused on subjects with low socioeconomic status and educational problems. Given that educational attainment is an important protective factor against stress-related disorders such as depression (16), it is important to investigate the effects of residential mobility in individuals with higher education. The majority of previous studies examined psychosocial outcomes and did not include biological measures. In this study, we used data from a community-based sample of young adults with middle and high socioeconomic status to examine psychiatry-relevant outcomes and their neurobiological correlates. Specifically, we hypothesized that moving in adolescence was associated with internalizing disorders, such as social anxiety and depression. Furthermore, lower social support, lower social status, abnormal amygdala-connectivity, and decreased serum BDNF concentration are supposed to be related to moving during this critical time period. We also hypothesized that multiple moves were associated with more pronounced behavioral problems and more abnormal biological markers than a single move (dose-response relationship).

## Methods

### Subjects

Subjects were recruited using advertisements in local newspapers and University blackboard webpages. The recruitment in the student environment, the mentally challenging study procedures and the requirement to be fluent in German language provided us with a sample of predominantly young adults with relatively high educational attainment. Subjects were included into the study only after full explanation of the goals of the study and the risks of the study procedures. The study and the written consent were approved by the local ethics committee (Kantonale Ethikkommission Zürich). Individuals with severe psychiatric disorders such as schizophrenia, bipolar disorder, alcohol and drug dependence, and acute eating disorders were excluded.

The study is part of an ongoing neuroimaging study. We included 234 subjects who underwent MRI scanning and provided data on residential mobility in their adolescence. Out of the 234 subjects, 68 were diagnosed with lifetime major depressive disorder (MDD), including subjects in partial or full remission. Table 1 displays the clinical characteristics of the study sample categorized by residential mobility.

**Table 1:** Sociodemographic characteristics and residential mobility.

	**No moves**	**One move**	**Two or more moves**	**All**	***p* (uncorr.)**
*n*	164	50	20	234	
Female (%)	107(65.2)	35(70.0)	16(80.0)	158(67.5)	0.378a
MDD (%)	42(25.6)	19(38.0)	7(35.0)	68(29.1)	0.199a
Age [y] [M, (SD)]	25.1(4.9)	25.0(4.3)	24.2(4.0)	25.0(4.7)	0.707b
Education [y] [M, (SD)]	14.1(2.7)	14.3(2.8)	13.2(2.8)	14.1(2.8)	0.296b
Own income [1000SFR] [M, (SD)]	21.8(29.3)	18.4(17.9)	20.5(21.3)	21.0(26.6)	0.726b
Parents' income [1000SFR] [M, (SD)]	133(89)	116(93)	98(101)	126(91)	0.313b
Proportion of divorced parents (%)	43(35.2)	13(34.2)	11(73.3)	67(38.3)	0.014a
Proportion of subjects in a partnership (%)	86(54.1)	23(46.0)	10(52.6)	119(52.2)	0.607a
Social status subject [self-rated, M, (SD)]	6.06(1.48)	5.88(1.64)	4.85(1.50)	5.91(1.55)	0.004b
Social status subject's mother, [rated by subject M, (SD)]	6.23(1.78)	6.04(1.77)	4.95(2.06)	6.08(1.80)	0.014b
Subjects with regular alcohol consumption (at least once a week, %)	66(40.2)	21(42.0)	9(45.0)	96(41.0)	0.909a
Subjects with regular drug consumption (mainly cannabis, at least once a month, %)	9(5.5)	3(6.0)	0(0.0)	12(5.1)	0.548a

*a, chisquare test; b, One-way ANOVA*.

### Clinical Assessments

Subjects underwent an extensive online survey. The Liebowitz Social Anxiety Scale (LSAS) (17), the Five-Factor Inventory (NEO-FFI) (18), the Obsessive Compulsive Inventory Revised (OCI-R) (19), and questions regarding sociodemographic characteristics, social status and social support were administered online before the MRI session. On the day of the MRI session, subjects filled out self-report questionnaires on acute psychiatric symptoms: Beck's Depression Inventory (BDI) (20) and Beck's Anxiety Inventory (BAI) (21). Psychiatric diagnoses were made based on the Structured Clinical Interview for DSM-IV (22). Adolescent residential mobility was assessed by a single question: “How many times did you move between the ages of 10 and 16 years (i.e., moves with change of school).”

### fMRI Data

All participants were scanned with a 3.0T GE Discovery MR750, using an 8-channel receive-only head coil. The same BOLD-fMRI sequence was acquired during an emotional faces task and for a resting state sequence (eyes open, fixation). For both BOLD-fMRI sequences, the same T2^*^-weighted EPI image parameters were used (38 slices of 64 x 64 voxels, resulting in a resolution of 3.75 x 3.75 x 3.2 mm; TR = 1,925 ms, TE = 32 ms, flip angle = 90°; axial slices were tilted 20° from the AC-PC line forward to reduce potential signal dropouts in the amygdalae). The images were all warped to the MNI template and then smoothed with a 5 mm FWHM isotropic kernel (SPM12 for Matlab; https://www.fil.ion.ucl.ac.uk/spm/software/spm12). For four subjects, both task and resting state fMRI datasets could not be used; for the task fMRI, an additional 16 had to be excluded, for the resting state fMRI, an additional 3. Reasons for exclusion of fMRI datasets included: movement artifacts (5), sleepiness, e.g., with under 80% correct responses in the task (6), problems seeing the stimuli (4), software problems in the scanner or stimulus presentation pcs (4) or administrative reasons (lack of time after taking out the subject in the middle of the session in one subject, due to an artifact from a small metal drawstring tip recognized only during the scanning session); we had to exclude data from one subject because of a mild abnormality. The VOIs for the amygdalae were hand-drawn on a T2-weighted image coregistered with the normalized EPI-template. The VOIs for the orbitofrontal cortex were used from the conn toolbox (see below).

The fMRI task was programmed as closely as possible to the experiment used by Etkin et al. (23). It included unmasked fearful, masked fearful, and neutral faces to examine amygdala responses to threat cues. After five dummy scans, 243 volumes were collected over a duration of 467.8 s. Stimuli were presented on MR-compatible goggles (Nordic Neuro-Lab) by Cogent (Cogent2000v1.32) running on Matlab 2011b. fMRI contrasts were calculated according to the onsets of the blocks (block design folded with standard HRF using an autoregression model). For the present study, only the contrast “fearful faces vs. neutral faces” was used. All six movement parameters, as well as white matter and CSF-activations, were regressed out.

Resting state functional connectivities were calculated with the conn toolbox (https://www.nitrc.org/projects/conn), version 15 g. We concentrated on the functional connectivities of left-amygdala-left orbitofrontal cortex and right amygdala-right orbitofrontal cortex.

### Serum BDNF Concentration

Serum samples were collected between 10 h 24 min and 18 h 18 min [mean 14 h 06 min, SEM 6 min; the time of sampling did not differ between groups, ANOVA (*p* = 0.512)] and stored at −80°C before assaying BDNF content. BDNF concentrations in the serum were assessed with an enzyme-linked immunosorbent assay (ELISA) kit (Biosensis® Mature BDNF RapidTM ELISA Kit: Human, Mouse, Rat; Thebarton, SA, Australia). As described in the manufacturer's protocol, the samples were diluted (1:100) and detection of BDNF was carried out on pre-coated mouse monoclonal anti-mature BDNF 96-well plates. Within 5 min after addition of the stop solution, the absorbance was measured in a microplate reader set at 450 nm and a correction wavelength set to 690 nm, to determine BDNF concentrations according to the standard curve. All assays were carried out in duplicate and means were calculated.

### Statistical Analysis

We used group comparisons (Oneway ANOVAs and subsequent Tukey tests) and categorized the variable “moves during adolescents” into three categories, namely, zero moves, one move, and two or more moves (as to be expected, the number of moves followed a Poissonian-like distribution with a higher number of moves becoming extremely rare).

ANOVAs were possible because most variables tested could be interpreted on an interval scale, followed Gaussian distributions and showed approximately equal estimated population variances. The influence of third variables were assessed with ANCOVAs with a single control variable to minimize the impact of non-linearities and statistical dependencies among the control variables [control variables tested were sex (tested by looking at the sexes separately), age, education, alcohol consumption, Marijuana consumption; the self-rated social status of the subject, the subject's father and the subject's mother were also tested]. We used a significance level of *p* = 0.05 as a threshold of a statistically meaningful result; for the correction of multiple comparisons, we used a Bonferroni-Holm correction (p(corrected)=p(uncorrected)^*^(n+1-k), where n is the number of independent comparisons and k is the number in the ranked list of results according to rising (uncorrected) *p*-values. For the psychiatric indices and the amygdala-orbitofrontal functional connectivities, we treated several measurements as one measurement, because they tended to be highly correlated.

To elaborate on the specificity of the main finding, subsequent ANCOVAs were used with an uncorrected *p* = 0.05 alpha error level.

## Results

Out of the 234 subjects included in the study, 164 did not move between ages 10 and 16, 50 moved once, and 20 moved twice or more than twice. As shown in Table 1, objective indicators of social status such as educational achievement, personal income, and parental income were not associated with adolescent moving (all *p* > 0.250). However, moving was associated with lower subjective social status (*p* = 0.004) and lower ratings of the subject on the social status of the subject's mother (*p* = 0.014).

**Figure 1:**
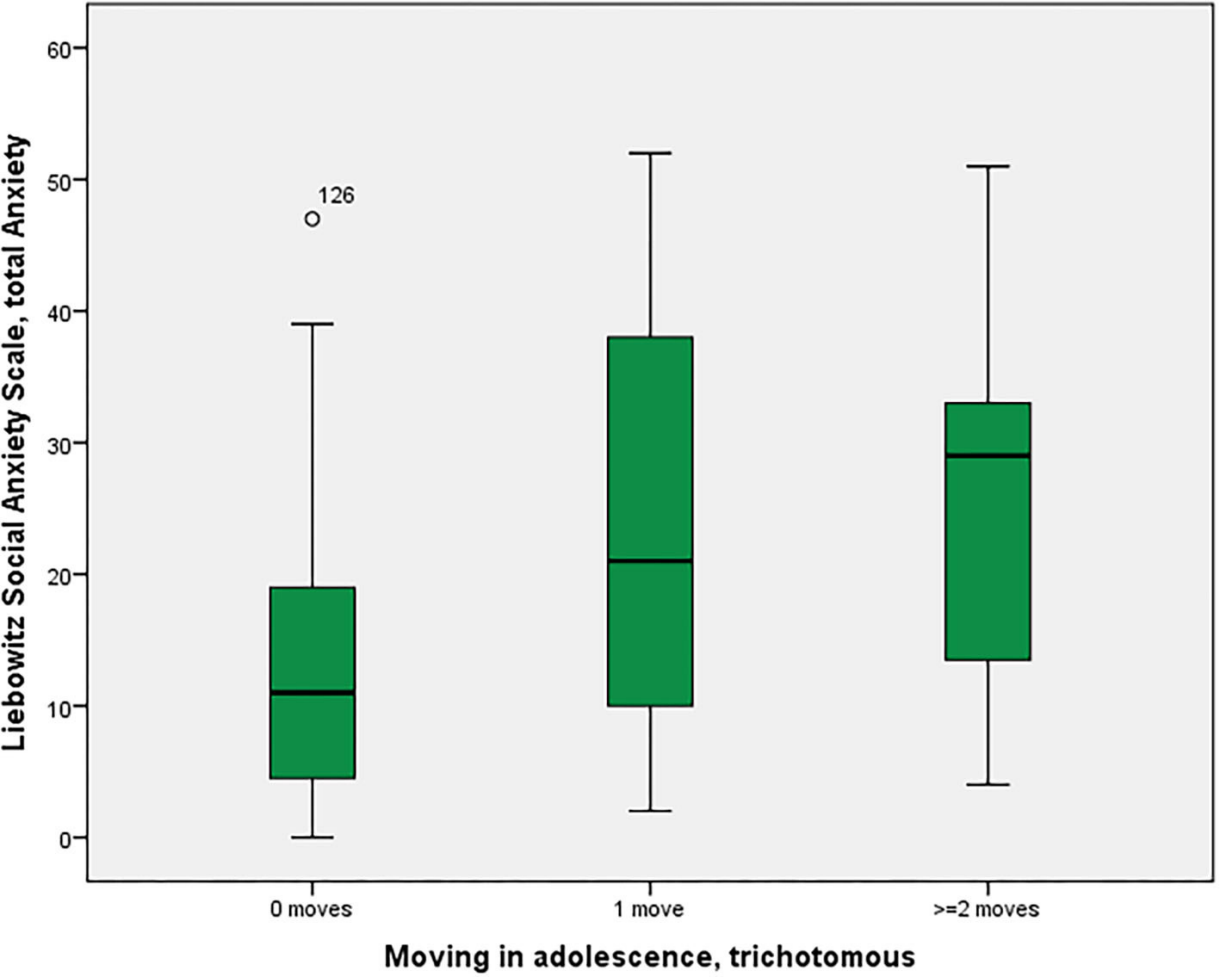
Residential mobility in adolescence and social anxiety in adulthood. Boxplot for the group comparison in social anxiety (Liebowitz Social Anxiety Scale, Anxiety) between subjects who did not move during adolescence (M = 13.4, SEM = 1.5, *n* = 52) with subjects who moved one time (M = 23.7, SEM = 4.7, *n* = 12) and twice or more than twice (M = 25.3, SEM = 6.2, *n* = 7). The ANOVA for simultaneous 3-group comparison is significant (*p* uncorrected = 0.007, with Bonferroni-Holm correction *p* = 0.021). Uncorrected *post-hoc* Tukey tests showed a significant result for zero moves vs. one move (*p* = 0.034), but not for the other comparisons (zero with two or more: *p* = 0.055; one with two or more: *p* = 0.960).

As shown in Figure 1, social anxiety as assessed using the LSAS was significantly associated with the three moving categories (*p* = 0.028 with Bonferroni-Holm correction) with non-movers having the lowest score of 13.4, followed by single-movers (23.7), and multi-movers (25.3) [in *post-hoc* Tukey tests, only the comparison 0 moves vs. 1 move turned out to be significant, p(uncorr) = 0.034]. Similarly, other anxiety/fear indicators also showed moving group differences, partly reaching significance (LSAS avoidance; BAI), partly as non-significant trends (neuroticism NeoFFI: OCD) (see Table 2).

**Table 2:** Detailed statistics for group comparisons.

	**No moves M(SEM)**	**One move M(SEM)**	**Two or more moves M(SEM)**	**ANOVA *p* (uncorr)**	**ANOVA *p* (corr)**	**Significant *post-hoc* tests (Tukey, *p* < 0.05 uncorr)**
LSAS anxiety	13.4(1.5)	23.7(4.7)	25.3(6.2)	0.007	0.021	0 vs. 1 mvs
LSAS avoidance	11.7(1.4)	24.6(4.6)	18.6(5.7)	0.004	0.012	0 vs. 1 mvs
Neuroticism NeoFFI	18.8(0.7)	20.9(1.2)	22.9(1.8)	0.066	0.198	
BAI	4.77(0.41)	5.45(0.82)	9.60(2.63)	0.005	0.015	0 vs. ≥ 2, 1 vs. ≥ 2
Obsessive Compulsive Inventory Revised (OCI-R)	8.80(0.59)	12.00(1.33)	9.85(1.87)	0.050	0.150	
Task fMRI BOLD-activation right amygdala	0.09(0.02)	0.07(0.03)	0.18(0.06)	0.118	0.118	
Functional connectivity left amygdala - left OFC	0.31(0.03)	0.47(0.07)	0.36(0.11)	0.082	0.164	
Functional connectivity right amygdala - right OFC	0.21(0.03)	0.42(0.06)	0.21(0.11)	0.008	0.016	0 vs. 1 mvs
BDNF (ng/ml)	12.0(0.4)	10.0(0.6)	8.3(1.3)	0.003	0.012	0 vs. 1 mvs

**Figure 2:**
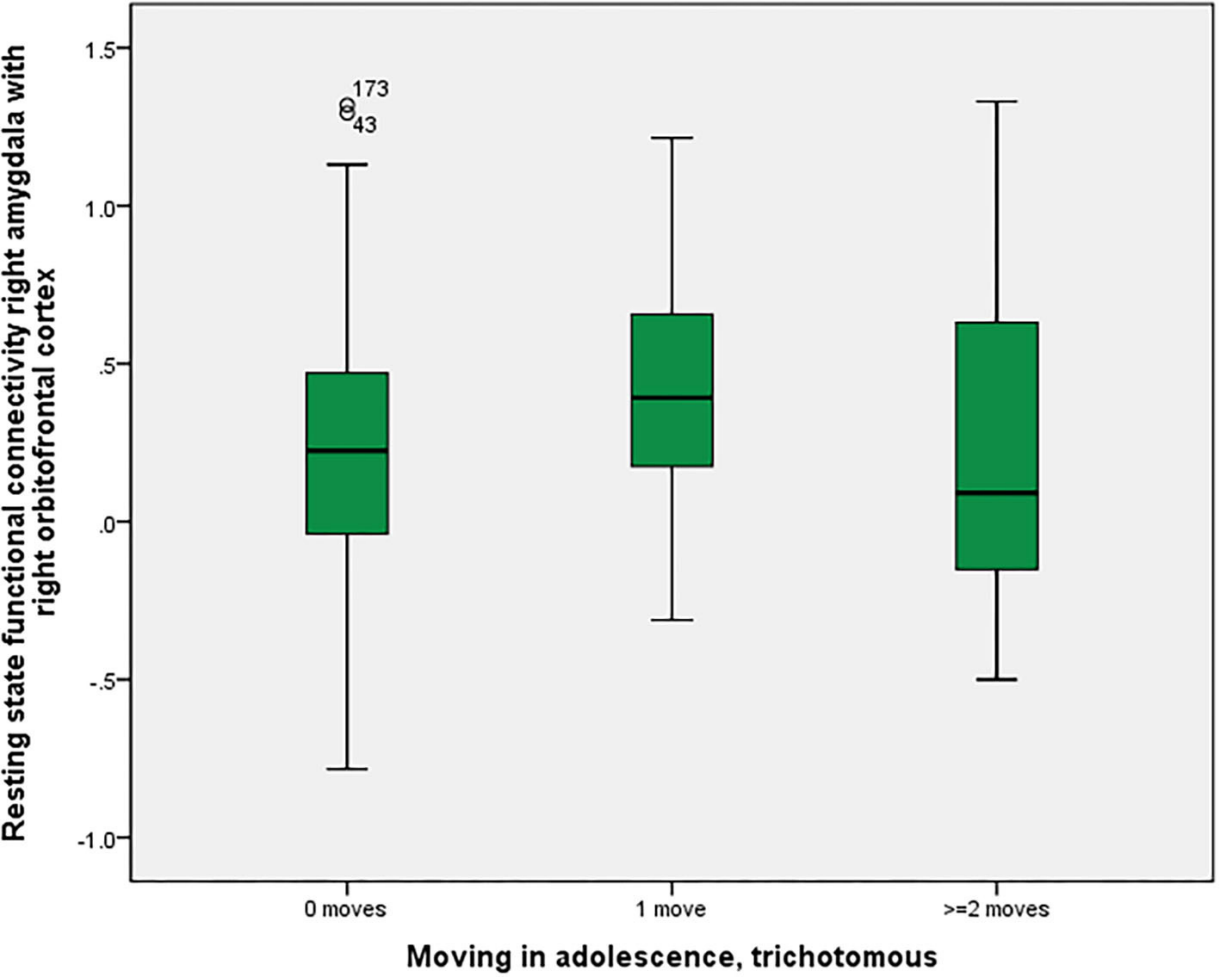
Residential mobility in adolescence and functional connectivity right amygdala—right orbitofrontal cortex. Boxplot for the group comparison in functional connectivity between right amygdala and right orbitofrontal cortex (qualitatively similar for other amygdala-orbitofrontal connectivities, e.g., left amygdala-left orbitofrontal cortex; beta-values corrected for scan-to-scan motion and activations in the white matter/CSF segments) between subjects who did not move during adolescence (M = 0.21, SEM = 0.03, *n* = 161) with subjects who moved one time (M = 0.42, SEM = 0.06, *n* = 41) and twice or more than twice (M = 0.21, SEM = 0.11, *n* = 20). The ANOVA for simultaneous 3-group comparison is significant (*p* uncorrected = 0.008, with Bonferroni-Holm correction *p* = 0.016). Uncorrected *post-hoc* Tukey tests showed a significant result for zero moves vs. one move (*p* = 0.006), but not for the other comparisons (zero with two or more: *p* = 0.999; one with two or more: *p* = 0.140).

As shown in Figure 2, there was a significant association between right amygdala-right orbitofrontal functional connectivity (*p* = 0.016 corrected with the Bonferroni-Holm method), between non-movers, single-movers and multi-movers. In response to suggestions from the reviewers, we calculated two MANOVAs, with the two “parallel” connectivities (left amygdala-left orbitofrontal cortex and right amygdala-right orbitofrontal cortex) or all four “parallel plus crossing” connectivities (left-left, right-right, left-right, right-left) as one dependent variable (all of them were significantly correlated) and in both cases found significant relationships with number of moves in adolescence (*p* = 0.001).

**Figure 3:**
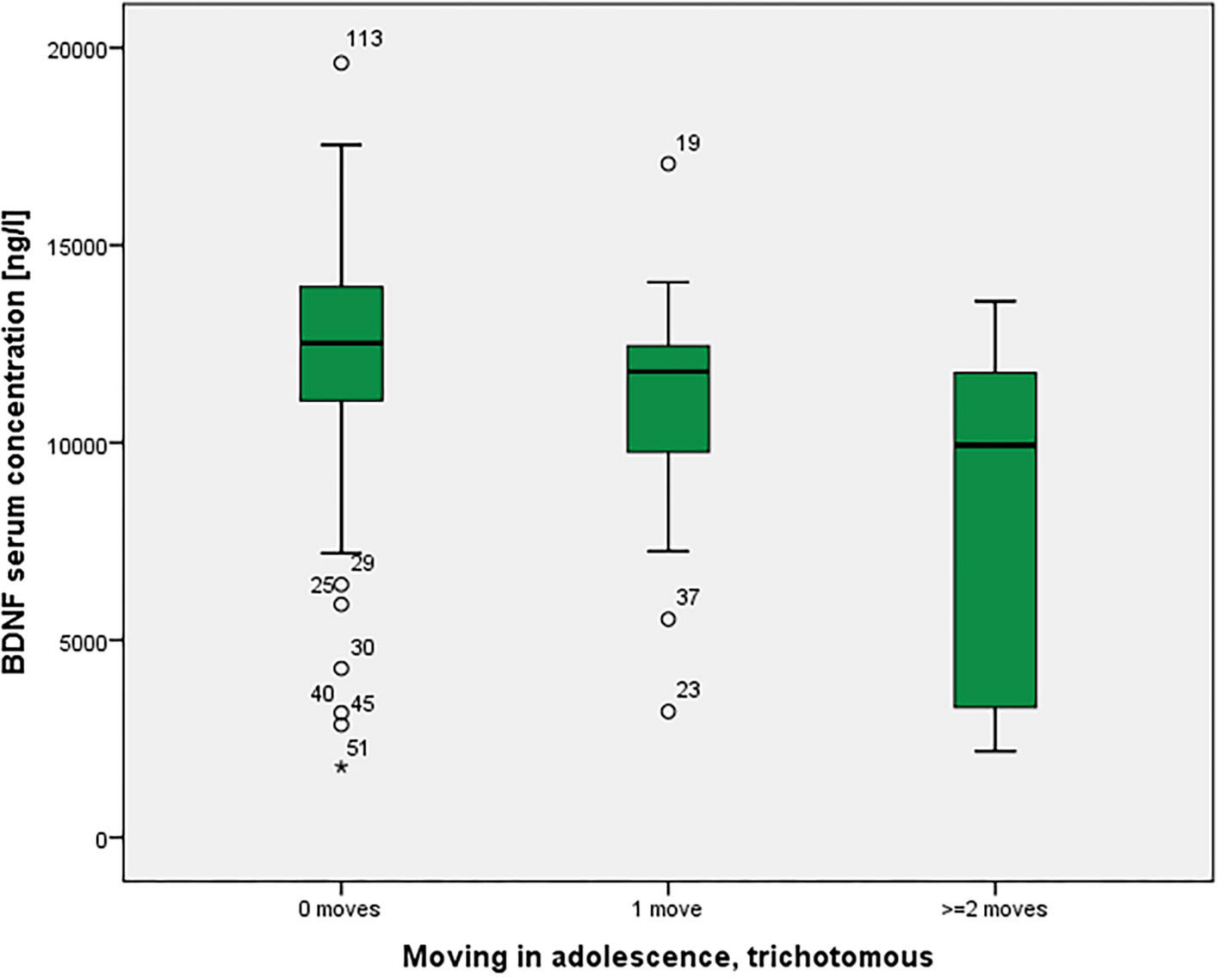
Residential mobility in adolescence and Brain-Derived Neurotrophic Factor (BDNF) in the serum. Boxplot for the group comparison in serum BDNF concentration (ng/l) between subjects who did not move during adolescence (M = 12.0, SEM = 0.4, *n* = 74) with subjects who moved one time (M = 11.0, SEM = 0.7, *n* = 21) and twice or more than twice (M = 8.3, SEM = 1.3, *n* = 11). The ANOVA for simultaneous 3-group comparison is significant (*p* uncorrected = 0.007, with Bonferroni-Holm correction *p* = 0.021). Uncorrected *post-hoc* Tukey tests showed a significant result for zero moves vs. two or more moves (*p* = 0.002), but not for the other comparisons (zero with one: *p* = 0.481; one with two or more: *p* = 0.072).

BOLD activations of the right amygdala in a fearful face task were not statistically significantly different among the groups (*p* = 0.118 corrected). As shown in Figure 3, there was a significant association between moving and serum BDNF concentrations (*p* = 0.021 with Bonferroni-Holm correction), with highest BDNF concentrations in non-movers, followed by single-movers and multi-movers. While anxiety/fear indices were all very strongly correlated, functional connectivity between right amygdala and right orbitofrontal cortex, and right amygdala activation and serum BDNF concentration were not correlated with each other, and not correlated with LSAS (see Table 3).

**Table 3:** Correlations between psychiatric indicators and physiological variables.

	**LSAS avoidance**	**Neuroticism**	**BAI**	**OCIR**	**Functional connectivity rAMY-rOFC**	**BDNF**
LSAS anxiety	**0.870**	**0.632**	**0.643**	**0.577**	(0.139)	(0.104)
LSAS avoidance		**0.606**	**0.554**	**0.622**	(0.056)	(0.012)
neuroticism			**0.581**	**0.510**	(−0.041)	(−0.019)
BAI				**0.490**	(−0.021)	(−0.112)
OCIR					(−0.035)	(−0.036)
Functional connectivity rAMY-rOFC						(0.121)

*Non-significant correlations in brackets; bold means p < 0.010*.

When controlling for the social status and income variables as linear covariates in ANCOVA, only self-rated social status substantially reduced the effect of the moving variable (e.g., on LSAS anxiety, reducing the eta-square effect size from 0.134 to 0.080; on BAI, reducing the eta-square effect from 0.046 to 0.023), income of the parents reduced the effect size of right amygdala activation (eta-square from 0.020 to 0.004) whereas years of education, own income, income of the parents or social status of the parents did not significantly affect any of the results. As shown in Table 1, drug and alcohol consumption were not associated with residential mobility.

## Discussion

In this study of young adults with an average education duration of 14 years, we found a positive relationship between moving during adolescence and social anxiety, anxiety symptoms as assessed with the BAI and low subjective status in adulthood. The examination of resilience-related biological measures pointed in the same direction: moving in adolescence was associated with low serum BDNF concentration and abnormal amygdala-orbitofrontal functional connectivity in adulthood.

There is increasing evidence from epidemiological and sociological research that compared with children, adolescents are more social, form more complex peer relations, and are more sensitive to acceptance and rejection by their peers. Social interactions have an important influence on mentalizing competences, such as perspective taking, that are still under development during adolescence (24). In this period, important neuronal reorganizations make the brain vulnerable to change. Adolescence is the peak age of stress-related disorders (7). Specifically, the plasticity of the connection between the amygdala and the prefrontal cortex is of crucial importance since it is substantially involved in affect regulation after puberty (10). As a result, impairments of social integration and brain development may have lasting consequences on mental health.

Twin studies estimate that around 70% of risk factors of social anxiety are non-genetic in nature (25), and that both familial and non-familial environmental factors play an important role in the development of social anxiety disorders. So far, there is a lack of knowledge of concrete, modifiable environmental risk factors in psychiatry. Residential mobility can be considered a modifiable risk factor that has both familial and non-familial aspects. Based on animal and human research, theoretical models assume that inadequate development of the perception of control is strongly implicated in the pathogenesis of excessive anxiety (26). It is plausible that not only parenting style, but also moving in adolescence can impair the sense of control.

Our finding of an association of moving with social status and social avoidance fits well into previous research. Families that frequently move may have a general lack of connections to their neighbors and communities and may have more difficulty building these connections (5). Movers have fewer friends and are socially more isolated (1). Our study adds to these findings, demonstrating that moving during adolescence can lead to social avoidance well into adulthood.

In our study, adolescent income and parental income were not associated with moving. This suggests that economic factors may not be the main driver of the association between residential mobility and mental health, confirming previous results that “upward” moves into higher quality neighborhoods have a comparable negative impact on adolescents as “downward” moves into poorer neighborhoods (6).

Educational attainment has been found to be a strong protective factor against stress-related disorders such as depression. A meta-analysis of 37 studies demonstrated a 3% decrease in the risk of depression for each additional year of education (27). This effect seems to be largely independent of the genetic risk of mood and anxiety disorders (16). Given that most studies on the health consequences of residential mobility have been conducted in subjects with low educational attainment, the observation from our study that moving may have a significant negative health impact even in individuals with higher education adds to the existing literature. Previous studies have reported an association between high residential mobility and the development of alcohol and other drug-related problems among adolescents and young adults (28). We did not find such an association, possibly because high educational attainment and economic security in our study subjects provided protective factors against substance abuse (29).

The amygdala has been found to act as a central structure in the association between peripubertal stress, adolescent brain development and later behavioral problems including social avoidance (30, 31). The amygdala is under the influence of top-down pathways from cognitive control regions in the orbitofrontal cortex (32). These pathways develop fully during adolescence, and are plastic and stress-sensitive. Thus, abnormal amygdala-orbitofrontal functional connectivity is a plausible mediator of the association between adolescent stress and later anxiety traits and disorders. Our finding of increased right amygdala-orbitofrontal connectivity in single-movers suggests a compensated state, in which increased connectivity reflects an increased effort of the prefrontal cortex to control right amygdala overactivity. One might speculate that in subjects with exposure to several moves during adolescence, compensation is insufficient, leading to more social anxiety. However, the frequent mover sample was small and interpretation of this finding remains highly speculative.

There is increasing evidence from preclinical studies that serum BDNF concentration mediates the negative consequences of social stress including social isolation on the pathways from the prefrontal cortex to the amygdala that regulate fear and other negative emotions. In rats, social isolation is consistently associated with decreased expression of BDNF (33). In humans, downregulation of BDNF has been related to mood and anxiety disorders, while high BDNF concentration has appeared to be a resiliency factor (34). BDNF may be particularly important in the fear circuit maturation during adolescence (13, 35). In our study, we found a negative dose-response-type relationship between moving during adolescence and serum BDNF concentrations in adulthood. This may reflect a consequence of the stress symptoms experienced by the participants who were exposed to moving during adolescence. Alternatively, low serum BDNF may reflect long-term consequences of moving, possibly mediated by stress-related epigenetic modifications (36). Interestingly, the perceived social status of the mother reduced the effect of moving on BDNF and social avoidance. This is in line with previous literature on social capital that emphasizes the important role of parents in mitigating losses of community social capital resulting from a family's moves (37).

The following limitations merit comment. This was a cross-sectional study where moving in adolescence was assessed retrospectively. Since the participants were young adults, moving in adolescence involved a change in school and was a relatively recent event (or events) that can be easily and reliably recognized. Since we did not assess a full moving history, we cannot analyze our data regarding critical phases of the psychobiological effects of moving. Social factors contributing to moving may have confounded the relationship between mobility and psychopathology. We included mainly highly educated people, which may reduce generalizability of the results to populations with low socioeconomic status. We assessed BDNF in blood. Because the relationship between brain and serum BDNF has not yet fully been elucidated, the interpretation of serum BDNF should be undertaken with caution.

In conclusion, this study demonstrated associations between moving during adolescence and increased social stress-sensitivity in adulthood, illustrated by increased social avoidance, abnormal amygdala-orbitofrontal functional connectivity and low serum BDNF concentration. The results of this study are clinically and socially relevant since moving during adolescence is a potentially modifiable risk factor of stress sensitivity. The exploratory finding that the perceived social status of the mother mitigated the effects of moving on BDNF encourages future research into protective factors on moving during adolescence.

## Data Availability Statement

The raw data supporting the conclusions of this article will be made available by the authors, without undue reservation.

## Ethics Statement

The studies involving human participants were reviewed and approved by Kantonale Ethikkommission Zürich. The patients/participants provided their written informed consent to participate in this study.

## Author Contributions

GH: designed study and wrote first draft of the manuscript. MH: collected data and helped setting up the study. SM: collected data. RT: provided imaging methods and designed the study. CR: collected data and wrote parts of the manuscript. AB: collected data, conceptualized and performed data and statistical analyses, and wrote first draft. All authors revised and approved the final version of the submitted manuscript.

## Conflict of Interest

The authors declare that the research was conducted in the absence of any commercial or financial relationships that could be construed as a potential conflict of interest.
